# Social Transfer of Pathogenic Fungus Promotes Active Immunisation in Ant Colonies

**DOI:** 10.1371/journal.pbio.1001300

**Published:** 2012-04-03

**Authors:** Matthias Konrad, Meghan L. Vyleta, Fabian J. Theis, Miriam Stock, Simon Tragust, Martina Klatt, Verena Drescher, Carsten Marr, Line V. Ugelvig, Sylvia Cremer

**Affiliations:** 1 Evolutionary Biology, IST Austria (Institute of Science and Technology Austria), Klosterneuburg, Austria; 2 Institute of Bioinformatics and Systems Biology, Helmholtz Center Munich, Neuherberg, Germany; 3 Evolution, Behaviour & Genetics, University of Regensburg, Regensburg, Germany; Stanford University, United States of America

## Abstract

Due to the omnipresent risk of epidemics, insect societies have evolved sophisticated disease defences at the individual and colony level. An intriguing yet little understood phenomenon is that social contact to pathogen-exposed individuals reduces susceptibility of previously naive nestmates to this pathogen. We tested whether such social immunisation in *Lasius* ants against the entomopathogenic fungus *Metarhizium anisopliae* is based on active upregulation of the immune system of nestmates following contact to an infectious individual or passive protection via transfer of immune effectors among group members—that is, active versus passive immunisation. We found no evidence for involvement of passive immunisation via transfer of antimicrobials among colony members. Instead, intensive allogrooming behaviour between naive and pathogen-exposed ants before fungal conidia firmly attached to their cuticle suggested passage of the pathogen from the exposed individuals to their nestmates. By tracing fluorescence-labelled conidia we indeed detected frequent pathogen transfer to the nestmates, where they caused low-level infections as revealed by growth of small numbers of fungal colony forming units from their dissected body content. These infections rarely led to death, but instead promoted an enhanced ability to inhibit fungal growth and an active upregulation of immune genes involved in antifungal defences (*defensin* and *prophenoloxidase*, *PPO*). Contrarily, there was no upregulation of the gene *cathepsin L*, which is associated with antibacterial and antiviral defences, and we found no increased antibacterial activity of nestmates of fungus-exposed ants. This indicates that social immunisation after fungal exposure is specific, similar to recent findings for individual-level immune priming in invertebrates. Epidemiological modeling further suggests that active social immunisation is adaptive, as it leads to faster elimination of the disease and lower death rates than passive immunisation. Interestingly, humans have also utilised the protective effect of low-level infections to fight smallpox by intentional transfer of low pathogen doses (“variolation” or “inoculation”).

## Abstract 2

Social contact with fungus-exposed ants leads to pathogen transfer to healthy nest-mates, causing low-level infections. These micro-infections promote pathogen-specific immune gene expression and protective immunization of nest-mates.

## Author Summary

Close social contact facilitates pathogen transmission in societies, often causing epidemics. In contrast to this, we show that limited transmission of a fungal pathogen in ant colonies can be beneficial for the host, because it promotes “social immunisation” of healthy group members. We found that ants exposed to the fungus are heavily groomed by their healthy nestmates. Grooming removes a significant number of fungal conidiospores from the body surface of exposed ants and reduces their risk of falling sick. At the same time, previously healthy nestmates are themselves exposed to a small number of conidiospores, triggering low-level infections. These micro-infections are not deadly, but result in upregulated expression of a specific set of immune genes and pathogen-specific protective immune stimulation. Pathogen transfer by social interactions is therefore the underlying mechanism of social immunisation against fungal infections in ant societies. There is a similarity between such natural social immunisation and human efforts to induce immunity against deadly diseases, such as smallpox. Before vaccination with dead or attenuated strains was invented, immunity in human societies was induced by actively transferring low-level infections (“variolation”), just like in ants.

## Introduction

### Immunological Memory at the Individual and Society Level

The first encounter of a host with a particular pathogen often leads to the outbreak of the disease, yet a secondary exposure rarely causes illness, due to the immunological memory of the host. Whereas immune memory in vertebrates is well appreciated [1], the phenomenon of an individual developing specific immunity against a subsequent pathogen exposure—referred to as immune priming—has only recently been described in invertebrates, both within the lifetime of an individual [2]–[8] and in transgenerational protection of offspring ([8]–[12], but see [13]). In contrast to vertebrates, the underlying mechanisms are not yet understood in invertebrates [14],[15]. In addition to this immunological memory at the level of individuals, a similar phenomenon occurs at the colony level in insect societies [16]–[18]. Society members act collectively, similar to cells in a body, and work as a superorganism [19],[20] in multiple aspects, including anti-pathogen defence [21]. For instance, an initial pathogen contact of a colony due to the presence of exposed individuals has been shown to lower the susceptibility of their nestmates to infection when they are later exposed to the same pathogen [16]–[18]. In addition to this physiological “social immunisation,” the collectively performed hygiene behaviour that complements individual defences in social insects [22]–[24] is also affected. Allogrooming of exposed individuals by their nestmates occurs more frequently in colonies with previous experience with this pathogen than in naive colonies [25],[26]. In contrast to individual immune priming, social immunisation thus refers to a protection of naive individuals of a colony after social contact to exposed individuals.

The phenomenon of social immunisation occurs broadly in insect societies—in unrelated social host species (ants and termites) and against divergent pathogen taxa (fungi [17],[18] and bacteria [16])—yet the mechanisms underlying this effect are largely elusive (but see [16]) and have only been hypothesised upon for fungal pathogens [3],[17],[18],[27]. In this study, we therefore aimed to determine the underlying causes of social immunisation in colonies of the ant *Lasius neglectus* after exposure of single individuals to the entomopathogenic fungus *Metarhizium anisopliae*, a common natural pathogen of ants [28],[29]. In this system, we have previously described that 5 d of social contact to an individual exposed to fungal conidia (conidiospores; [30]) led to a lower susceptibility of nestmate ants when challenged with a high fungal dose after this period [18]. It remained open, however, which social interactions may trigger this effect and how they elicit changes in nestmate immunity.

### Potential Routes to Social Immunisation

The observed protection in nestmates of exposed ants may be caused by the active upregulation of their own immune systems following social contact to the fungus-exposed individual. Alternatively, social transfer of immune mediators produced by colony members may lead to passive protection of nestmates without requiring the activation of their own immune systems (as outlined by [3],[17],[27]). The active and passive route to social immunisation may also act in concert.

Active upregulation of the nestmates' immune system may be caused by perception of a trigger signal elicited from the exposed individual, possibly of behavioural or chemical nature. In humans, mere visual perception of sick individuals was recently shown to cause preventive stimulation of the immune system [31]. Similarly, in plants, herbivory defence was promoted by perception of volatile chemical cues elicited by an attacked neighbouring plant [32]. Active stimulation of the immune system can also be caused by low-level infections [3],[8],[33],[34], which may result from social transfer of the pathogen from the exposed individual to its nestmates (as suggested by [3]), occurring during “normal” social interactions, or as a byproduct of collective sanitary behaviour such as allogrooming of the exposed individual by its nestmates [22],[35].

Passive immunisation may result from a social exchange of antimicrobials produced by the exposed individuals and transferred to their nestmates. Possible transfer pathways include the “external route” over the body surface or the “internal route” by exchange of body fluids [16]. The external body surface (cuticle) of ants is covered with antimicrobial substances produced in an ant-specific gland (metapleural gland [36],[37]) and nestmates could easily pick up these substances and apply them on their own bodies by allo- and self-grooming. Immune effectors produced inside the body of infected individuals may be exchanged during the common social feeding behaviour of regurgitation and feeding of trophallactic droplets [16],[38], as has recently been suggested as a mechanism for social immunisation of ant colonies after bacterial exposure [16]. Whereas bacterial infections are typically orally transmitted [39], entomopathogenic fungi are externally transmitted, making distinct disease dynamics of these pathogen taxa likely.

In this study, we applied a multi-level approach to determine the functional mechanism of social immunisation of ant colonies against a fungal pathogen. We analysed the behavioural interaction rates between group members and determined whether social contact may lead to exchange of the pathogen or immune effectors, or whether social immunisation may be triggered by social signals. We determined both the physiological immunity of fungus-exposed individuals and their nestmates, as well as their immune gene expression. Lastly, we developed an epidemiological model to explore long-term colony-level effects of social immunisation depending on the underlying mechanisms.

## Results and Discussion

### Nestmates of Fungus-Exposed Ants Show Increased Antifungal Defence

**Figure 1:**
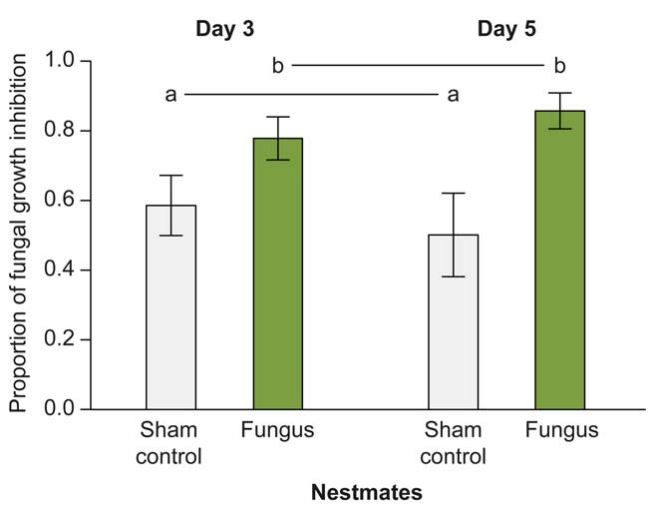
Antifungal immune assay of nestmates after social contact to treated individuals. Nestmates of fungus-exposed individuals (light green bars) inhibited fungal growth significantly more than nestmates of control-treated individuals (light grey bars), both after 3 and 5 d of social contact with the exposed ant. Bars indicate mean ± SEM of proportional antifungal activity compared to the growth control (*n* = 10 samples per treatment consisting of a pool of five individuals each). Different letters indicate statistically significant differences at α = 0.05.

We have previously shown that social contact to a *Lasius* worker exposed to conidia (dispersal form, conidiospores; [30]) of the entomopathogenic fungus *M. anisopliae*, but not to control-treated ants, increased the survival of previously naive nestmates when challenged with the same *M. anisopliae* strain 5 d later [18]. We now directly assessed the immune function of nestmates with a novel and sensitive “antifungal activity assay.” We incubated ant tissue with blastospores (within-host infection form; [30]) of the fungus to measure the ability of ants to inhibit fungal growth. We found a significantly higher antifungal activity in nestmates of fungus-exposed as compared to nestmates of control-treated individuals (Figure 1). This was true not only after 5 d of social contact to an exposed individual, but already after 3 d (GLM, *F* = 3.859, *df* = 3, *p* = 0.017; treatment type [fungus treatment versus sham control]: *F* = 10.634, *df* = 1, *p* = 0.002; time [3 versus 5 d post-treatment]: *F* = 0.001, *df* = 1, *p* = 0.973; interaction [Treatment Type×Time]: *F* = 0.942, *df* = 1, *p* = 0.338).

To understand the mechanism behind increased antifungal defence in nestmates of exposed ants, it is important to study the behaviour of group members. First, behavioural changes of individuals after fungal exposure may be a signal to their nestmates to upregulate their immune system. Second, the social interactions define the routes and opportunities for potential exchange of immune effectors [40],[41] or the pathogen itself [42].

### Behaviourial Interactions as Pathways for Pathogen Exchange Among Colony Members

Compared to control-treated ants, which did not elicit social immunisation in their nestmates, fungus-exposed ants did not show significantly changed rates of either brood care behaviour [18] or self-grooming activity (LVU, unpublished data). Similarly, other studies found that pathogen exposure had no effect on self-grooming [26] or only when doses present in the colony were very high [25]. This makes it unlikely that nestmates may have perceived a trigger signal by social interaction or potential observation of the individual behaviour of exposed ants.

To obtain information on possible pathways for transfer of the pathogen or immune mediators, we analysed the social interactions between colony members in more detail. As in our original experimental setup we grouped five naive nestmates with a single treated *Lasius* worker that had either received infectious *M. anisopliae* conidia (fungus treatment) or the same treatment without the pathogen (sham control). We observed three types of social interactions between group members. Antennation behaviour—that is, nestmate recognition behaviour by antennal contact [43]—occurred extremely rarely (6.6% of all interactions). Moreover, rates did not differ between treated and nestmate ants or among nestmates, for both fungus treatment and sham control (Generalised Linear Model [GLM] with negative binomial errors, LR *χ^2^* = 1.969, *df* = 3, *p* = 0.579; data not shown). All other social interactions observed between group members consisted of (a) allogrooming (i.e., cleaning the body surface of another ant) and (b) trophallaxis behaviour (i.e., exchange of regurgitated liquid food droplets) [43]. Both may be important pathways for social immunisation [3],[16],[17],[27].

It is well known that nestmates actively contact exposed individuals and remove infectious material with their mouth by allogrooming, which is a very efficient social sanitary behaviour [43],[44] increasing survival of pathogen-exposed individuals, but typically not compromising the survival of the nestmates [25],[35],[45],[46]. Still, the grooming ant may contract the pathogen if it is not able to kill all infectious material in its mouth (infrabuccal pockets; [47],[48]) or gut [49], or if it unintentionally rubs off conidia with other body parts than the mouth during this intimate social interaction. In addition, allogrooming may lead to uptake of antimicrobial substances from the body surface of an exposed individual similar to exchanges of cuticular waxes important for nestmate recognition [50].

**Figure 2:**
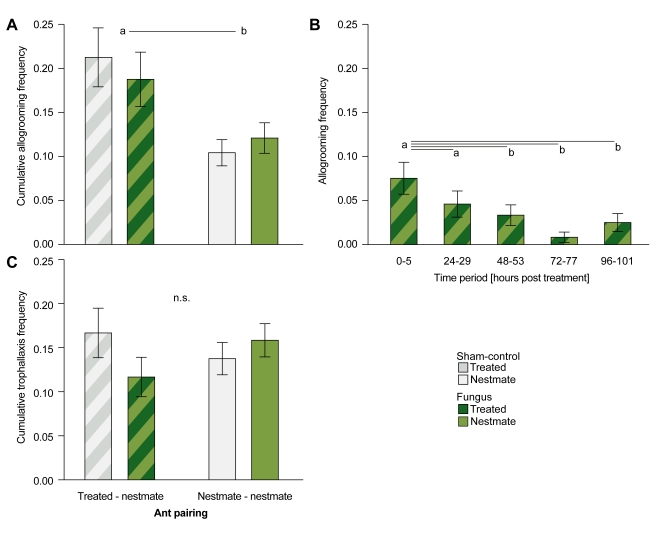
Behavioural interactions among group members. (A) Cumulative allogrooming frequencies over the 5 experimental days were significantly higher between treated individuals and their nestmates (striped bars, *n* = 240 per treatment type) than among nestmates (single colour bars, *n* = 480 per treatment type)—irrespective of treatment type (sham control, grey; fungus treatment, green). (B) Allogrooming frequencies between fungus-exposed individuals and their nestmates were significantly higher in the first 2 d of the experiment (observations 0–5 h and 24–29 h post-treatment) than at later time points (>48 h). (C) Cumulative frequencies of social feeding (trophallaxis behaviour) were not affected by type of group member and fungus versus control treatment. Bars represent average frequency (mean ± SEM) of interactions per individual over the total time (A and C) or periods (B) of observation. Different letters indicate statistically significant differences at α = 0.05; n.s., non-significant.

In our experiment, allogrooming rates between treated individuals and their nestmates were higher than among nestmates, but independent of the treatment type (fungus versus sham control; Figure 2A; GLM with negative binomial errors, LR *χ^2^* = 15.134, *df* = 3, *p* = 0.002; ant pairing [treated-nestmate versus nestmate-nestmate]: Wald *χ^2^* = 14.501, *df* = 1, *p*<0.001; treatment type [fungus versus sham control]: Wald *χ^2^* = 0.006, *df* = 1, *p* = 0.939). Upregulation of grooming frequency not only against individuals treated with infectious material but also with sham control solutions is known from previous studies [29],[51] and indicates that ants are very sensitive to applications on the bodies of their group members.

Despite the lack of difference between the two treatment types, intensive grooming towards treated individuals provides a potential route for transfer of either the pathogen itself or external immune effectors. One important factor is the timing of allogrooming expression during the infection course of *M. anisopliae*. Entomopathogenic fungi like *M. anisopliae* infect their hosts by external adhesion onto and active penetration of the cuticle [52]. After contact to the insect cuticle, the conidia first adhere loosely to the body surface within several hours and then germinate and form a penetration plug to actively enter the host body within approximately 24 to 48 h [46],[53]. Infection of the host and onset of an active immune response therefore occurs with a time delay of 2 to 3 d after exposure [54],[55]. Allogrooming in the first 1 to 2 d would therefore allow for pathogen transfer, whereas after this time exposed ants lose their infectiousness [26]. Intensified allogrooming 3 or 4 d after exposure would instead indicate exchange of external antimicrobial substances.

We analysed the time course of allogrooming frequency between treated individuals and their nestmates and found no change over time in the control treatment (GLM with repeated measures, time: *F* = 0.973, *df*_Huynh-Feldt_ = 3.648, *p* = 0.416). Allogrooming between nestmates and fungus-exposed individuals, however, was significantly higher in the first 2 d compared to later phases of the experiment (Figure 2B; time: *F* = 4.006, *df*_Huynh-Feldt_ = 3.306, *p* = 0.006 [day1 versus day2: *p* = 0.178; day1 versus day3: *p* = 0.041; day1 versus day4: *p* = 0.001; day1 versus day5: *p* = 0.014]). Based on these data we suggest that if a transfer between group members occurs via allogrooming, it more likely involves a transfer of conidia, detachable early after exposure, than immune effectors, which can only be upregulated and transferred to the cuticle after infection of the individual 24–48 h after exposure.

Social feeding via regurgitation and transfer of a trophallactic droplet may promote transfer of internal antimicrobial substances [16]. However, we found no differences in the rates of trophallaxis among all four groups, that is, neither between treated ants and their nestmates nor among the nestmates in either the fungus treatment or the control group (Figure 2C; GLM with negative binomial errors, LR *χ^2^* = 2.555, *df* = 3, *p* = 0.465). Our data show that fungal exposure does not alter trophallaxis rates between exposed individuals and their nestmates, making passive immunisation by transfer of internally produced antimicrobial substances rather unlikely in our model system. Our findings after fungal exposure contrast with observations that trophallaxis rates between individuals injected with dead bacteria or bacterial cell wall components (but also wounding controls) were increased compared to trophallaxis rates among untreated individuals ([16],[56], but see [57]).

Taken together, our behavioural observations strongly suggest exchange of the fungal pathogen between the fungus-exposed ant and its nestmates during intensified, early grooming as the most likely mechanism for the observed anti-fungal protection in the nestmates. We therefore determined if fungal conidia indeed were transferred from the exposed individual to its untreated nestmates by direct tracing of fluorescently labelled conidia.

### Pathogen Transfer to Nestmates Occurs After Social Contact to an Exposed Ant

We applied conidia of *M. anisopliae* labelled with red fluorescent protein (RFP) onto the exposed ant and determined their presence or absence on the cuticle of all group members after 2 d of social contact. We expected maximum pathogen transfer to have occurred at this time as (a) grooming activity between exposed ants and their nestmates is most intense in the first 30 h (Figure 2B) and (b) conidia are no longer transferable after this time [26],[53].

As expected we found high amounts of conidia on all directly exposed individuals (15/15) and furthermore detected low numbers of conidia on the cuticles of 37% (17/45) of nestmates (Figure S1; for negative controls see Materials and Methods). Interestingly, not only the quantity but also the location of conidia differed: whereas directly exposed individuals carried them mostly in areas likely difficult to reach by grooming such as joints and the antennal grooves, conidia on nestmates were rather attached to antennae and legs (Figure S1), suggesting that nestmates pick up the pathogen from the fungus-exposed individual during grooming. We can thus confirm pathogen transfer to the nestmates. In a next step we determined if the transferred conidia successfully established an infection in the nestmates.

### Fungus Transfer Leads to Sublethal Low-Level Infections in Nestmates

To quantitatively determine the infection load of directly fungus-exposed individuals and their nestmates over the course of the experiment, we sterilised their body surface to destroy all remaining conidia, dissected the ants, and plated their body contents on agar plates to count emerging fungal colony forming units (CFUs). We used morphological determination, as well as PCR [58], to confirm that outgrowing CFUs were indeed *M. anisopliae*, which was the case for all CFUs (see Figure S2 as an example). None of the 30 negative controls (see Materials and Methods) and none of the individuals measured within 24 h after exposure (0/10 fungus-treated, 0/14 nestmates; Figure S3) showed fungal growth, confirming that we effectively sterilised the ants and measured only live fungus from inside the body.

**Figure 3:**
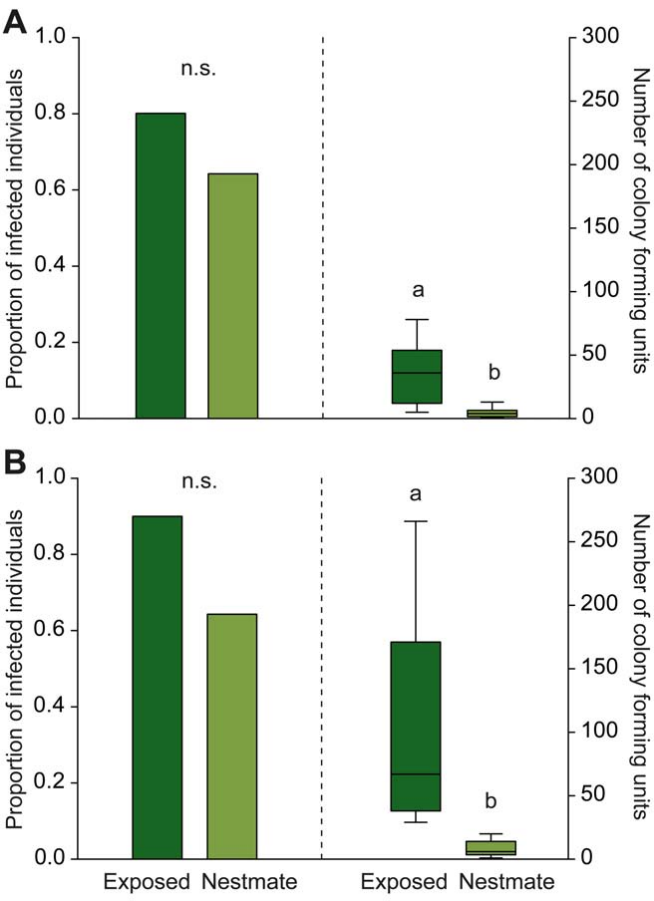
Fungal infection levels of treated individuals and their nestmates. Proportion of exposed individuals (dark green) and nestmates (light green) that show fungal growth inside their bodies (left panels) and number of fungal colony forming units in infected ants (right panels), after (A) 3 d and (B) 5 d of social contact. On both days, the proportion of infected individuals was equally high between directly fungus-exposed ants and their nestmates, indicating a high frequency of pathogen transfer between group members. Yet the infection load of infected nestmates was significantly lower on both days (approximately 8 times lower on day 3 and 12 times lower on day 5). Bars give the proportion of infected individuals in the different groups (*n* = 10 for directly exposed and *n* = 14 for nestmates per day) and boxplots show median and 25%–75% quartiles of CFUs in infected individuals (day 3: *n* = 8 directly exposed individuals and *n* = 9 nestmates; day 5: *n* = 9 each for directly exposed and nestmate ants). Different letters indicate statistically significant differences at α = 0.05.

Three as well as five days after exposure, CFUs grew from the body content of nearly all directly exposed ants (80% [8/10] and 90% [9/10]) and a similarly high number of nestmates (64% and 64% [each 9/14]; Figures 3, S3; Fisher's exact test; day 3, *p* = 0.653; day 5, *p* = 0.341). These data show that fungal infections in nestmates were more common than estimated from external pathogen transfer using labelled conidia. This may either indicate that we did not detect all conidia or that an additional infection route via the infrabuccal pocket in the mouth or the gut system occurred, for instance if groomed-off conidia were not completely prevented from germinating [47]–[49]. Fungal infection load in nestmates revealed that their infections were “low-level infections.” The number of CFUs growing out of their bodies when infected was significantly lower than those growing from directly exposed ants at both day 3 (Figures 3A, S3; Mann-Whitney U-test: *n_1_* = 8, *n_2_* = 9, *U* = 4.0, *p* = 0.002) and day 5 (Figures 3B, S3: *n_1_* = 9, *n_2_* = 9, *U* = 0.0, *p*<0.001). On average, the infection load of infected nestmates was 8 (4.4 versus 36.0) and 12 (8.1 versus 102.4) times smaller than that of directly exposed individuals on days 3 or 5, respectively.

Even if low-level infections occurred in the majority of nestmates, only 2% (3/150) died from a *M. anisopliae* infection after 5 d of social contact with the exposed individuals (who showed death rates of approximately 50% due to application of an LD_50_). This confirms that the effects of *M. anisopliae* infections are highly dosage dependent ([35] and MKo and STr, unpublished data).

### Low-Level Infections Are Sufficient to Explain the Increased Antifungal Activity of Nestmates

**Figure 4:**
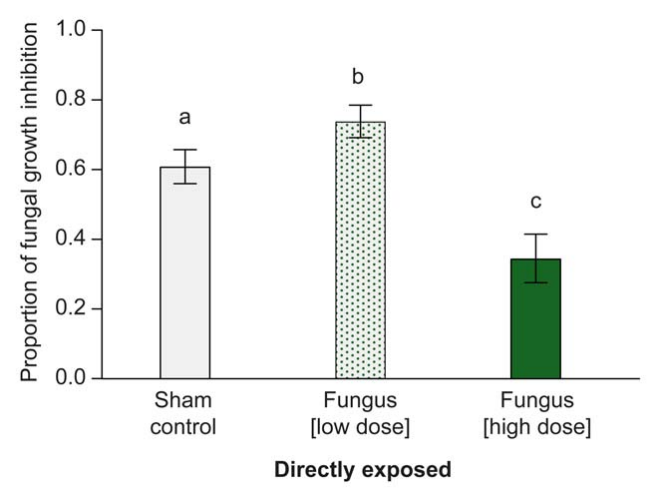
Antifungal activity of directly exposed individuals with low-level infections versus high-dose infections. Individuals directly exposed to a low pathogen dosage (exposure to LD_2_; dotted bar) had a significantly higher capacity to inhibit fungal growth than control-treated individuals (grey), whereas individuals exposed to a high dosage (exposure to LD_50_; green) had a significantly lower antifungal activity than controls and low-dose exposed ants (*n* = 10 for all groups). Bars show mean ± SEM of proportional antifungal activity compared to the growth control (*n* = 10 samples per treatment, each consisting of a pool of five individuals each). Different letters indicate statistically significant differences at α = 0.05.

To determine if the observed increase in antifungal activity of nestmates was a direct cause of these low-level infections, we established low-level infections in individuals in the absence of social interactions. To this end, we exposed isolated ants with a conidia dose that led to the same death rate (LD_2_) and infection level as observed in the socially exposed nestmates. We found that low-dose, directly exposed ants had a significantly increased antifungal activity 3 d after exposure compared to control-treated ants (Figure 4). Interestingly, directly exposed individuals with a high dose (LD_50_; as used for exposure of the single ants in our experiment above) showed a significantly decreased capacity to inhibit fungal growth (Figure 4; ANOVA: *F* = 10.361, *df* = 2, *p*<0.001; post hoc Protected Fisher's LSD tests all pairwise: sham control versus LD_2_: *p* = 0.046, sham control versus LD_50_: *p* = 0.021; LD_2_ versus LD_50_: *p*<0.001). This immune-suppressive effect of a high-dose infection is likely caused by the immune-interference and toxicity of *M. anisopliae* or by the fact that the immune responses had been depleted [41],[59]–[61]. Immune stimulation of low-level infections has previously been described for both vertebrates and invertebrates [3],[8],[33],[34], and its protective effect yielded clinical application in humans [62],[63] and poultry health management [64].

We have established that low-level infections, caused by social contact or direct low-dose exposure, lead to increased antifungal activity. Yet this does not exclude that nestmates with social contact to an exposed individual may also obtain signals that could actively trigger their antifungal immunity (similar to [31],[32]). To test this, we performed a “spatial-separation experiment” in which body contact and pathogen transfer to the exposed individual were prevented, whereas exchange of visual signals or volatile chemicals was still possible. The antifungal activity of nestmates of fungus-exposed individuals did not differ from that of nestmates of control-treated ants after 3 d of this constrained contact (*t* test: *t* = −0.376, *df* = 18, *p* = 0.711). These data suggest that a visual or volatile signal alone—at least one that acts over distance—is not sufficient to promote antifungal activity in the nestmates. Non-volatile chemical signals, such as cuticular hydrocarbons [65] that are part of the ants' cuticle, may in theory still play an additional role. However, their perception would always require body contact, which promotes pathogen transfer at the same time. We conclude that low-level infections alone provide a sufficient explanation for an active social immunisation of nestmates. We then tested if it may be complemented by a passive transfer of antimicrobial substances among nestmates.

### Passive Transfer of Antimicrobial Substances Is Unlikely

**Figure 5:**
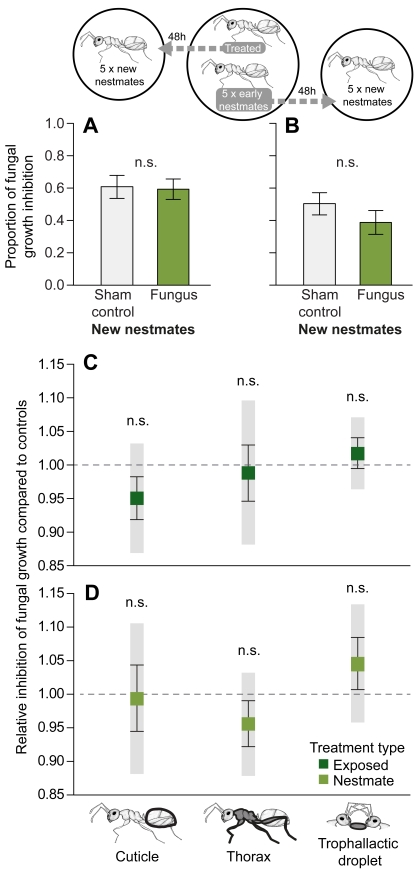
Antifungal activity measures to test for passive transfer of antimicrobial substances. (A, B) Antifungal activity of “new nestmates” of (A) directly treated ants and (B) early nestmates (*n* = 10 samples per group, each sample consisting of a pool of five individuals) for sham control (light grey) and fungus treatment (light green). The groups did not differ from one another. Bars show mean ± SEM of proportional antifungal activity compared to the growth control; n.s., non-significant. (C, D) Antifungal properties of the exterior and interior of fungus-exposed individuals compared to control individuals for the directly treated ants (C) and their respective nestmates (D). We found no difference in the potentially transferable substances from the body surface (cuticle of the ant gaster) and the thorax including the antimicrobially active metapleural glands, nor the trophallactic droplet between individuals treated with a sham control, or with the fungus (dark green for directly exposed individuals, light green for their nestmates). The antifungal activity of control-treated individuals (respectively, their nestmates) is given as a dotted line. Boxplots with whiskers represent mean ± SEM proportion and 95% confidence intervals (indicated in grey shading) of fungal growth inhibition of the ants from the fungus treatment, all standardised to the sham control (*n* = 10 samples per treatment, except for cuticle and thorax samples: *n* = 6 per group; each sample consisted of a pool of 5 ants); n.s., non-significant.

We performed a “temporal-separation experiment” and allowed the exposed ant to interact with its nestmates for 48 h. In this period, the pathogen (a) lost its ability to be transferred (for confirmation see Materials and Methods) and (b) established an infection in the ants, likely triggering an immune response [53]–[55]. After this time, we separated the treated individual and its “early nestmates” and added five “new nestmates” to both (see Figure 5A,B). Three days later, we measured the antifungal activity of the new nestmates. We found no difference between new nestmates of control-treated versus fungus-exposed ants (Figure 5A; *t* test: *t* = −0.159, *df* = 18, *p* = 0.876) or between new nestmates of early nestmates to a control-treated versus exposed individual (Figure 5B; *t* test: *t* = −1.273, *df* = 18, *p* = 0.219). This reveals that nestmates do not show an increase in antifungal activity if pathogen transfer is excluded.

Passive transfer of antimicrobials among the group members thus seems very unlikely as an explanation for social immunisation. However, such transferable substances might be upregulated in infected individuals and simply failed to elicit immunisation of nestmates in our experiment. We therefore also analysed both the fungus-exposed ant and its nestmates directly for the presence of potentially transferable antimicrobials 3 d after treatment. Although allogrooming rates among nestmates were low in both sham control and fungus-treated groups (Figure 2A), and trophallaxis rates were completely independent of treatment (Figure 2C), infected nestmates may be important in transferring antimicrobial substances, as their antifungal activity is higher than that of directly exposed ants, which suffer a much higher infection level (Figure 4).

We tested whether transferable substances of fungus-exposed individuals or their nestmates had higher antifungal activity than those of control-treated individuals and their respective nestmates. For externally transferable substances via allogrooming, we measured the antifungal activity of (a) the cuticle and (b) the thorax containing the metapleural gland content, which is known to have antimicrobial function and to be secreted onto the cuticle [36]. We also measured the antifungal activity of (c) the trophallactic droplet that is produced in the ant's body and is transferred via social feeding. We found that neither the cuticles nor the thoraxes containing the metapleural gland nor the trophallactic droplets of fungus-exposed individuals showed a different antifungal activity than the respective body parts of control-treated individuals (Figure 5C; *t* tests; cuticle: *t* = 1.064, *df* = 10, *p* = 0.312; thorax: *t* = 0.224, *df* = 10, *p* = 0.828; trophallactic droplets: *t* = −0.594, *df* = 18, *p* = 0.560). The same was true for the nestmates (Figure 5D; *t* tests; cuticle: *t* = 0.107, *df* = 18, *p* = 0.916; thorax: *t* = 0.894, *df* = 18, *p* = 0.383; trophallactic droplets: *t* = −0.717, *df* = 18, *p* = 0.482). This result was not an artifact caused by a potential effect of the control treatment, as the antifungal activity in these individuals was not different from completely untreated ants (Materials and Methods).

Taken together, we found no evidence for (a) a potential protective effect of nestmates in the absence of pathogen transfer and (b) potential upregulation of socially transferable antimicrobials in exposed colonies. This contrasts observations that trophallactic droplets obtained from bacteria-exposed ants had higher antibacterial activity than that of controls [16], making passive immunisation a likely mechanism involved in social immunisation of ant colonies after bacterial exposure [16], but not after fungal exposure. Instead, we documented that social interaction, most likely allogrooming, leads to pathogen transfer and sublethal low-level infections in the majority of nestmates of fungus-exposed individuals and that low-level infections are necessary and sufficient to induce an increased antifungal activity.

### Nestmates Show Active Upregulation of Immune Genes Specific for Antifungal Defence

To directly assess the effect of low-level infections on the immune response, we measured immune gene expression in nestmates using quantitative real-time PCR. We chose three immune genes known to be involved in the humoral and cellular defences of ants: (1) the antimicrobial peptide (AMP) *defensin* [66],[67], a soluble mediator that most closely resembles termicin, an antifungal peptide in termites [68],[69]; (2) *prophenoloxidase* (*PPO*), a key mediator of immune function in ants [70],[71] that is essential for the process of melanization upon infection by a variety of pathogens, including entomopathogenic fungi [72],[73]; and (3) *cathepsin L*, a lysosomal protease expressed in hemocytes [74], which has both antibacterial [75] and antiviral activity [76], but has not been implicated in antifungal responses. In *Camponotus pennsylvanicus*, another *cathepsin* (*cathepsin D*) was found to occur in higher amounts in the trophallactic droplets of ants after injection of heat-killed bacteria or LPS [16], suggesting the involvement of *cathepsins* in antibacterial responses in ants. We confirmed that our host ant, *L. neglectus*, also responds to bacterial infection with *cathepsin* upregulation. Septic injury with *Bacillus thuringiensis* led to upregulation of *cathepsin L* gene expression, but not *PPO*, or *defensin* expression, compared to pricked controls (Figure S4; *defensin*: *t* test; *t* = 0.186, *df* = 4, *p* = 0.862; *PPO*: *t* test; *t* = −1.448, *df* = 4, *p* = 0.221; *cathepsin L*: *t* test; *t* = −3.695, *df* = 4, *p* = 0.021; gene expression standardised to the housekeeping gene *18s rRNA*). The choice of these three immune genes in this study therefore allowed us to examine the specific effects of social immunisation against the fungus *M. anisopliae* on immune pathways involved in insect defences.

**Figure 6:**
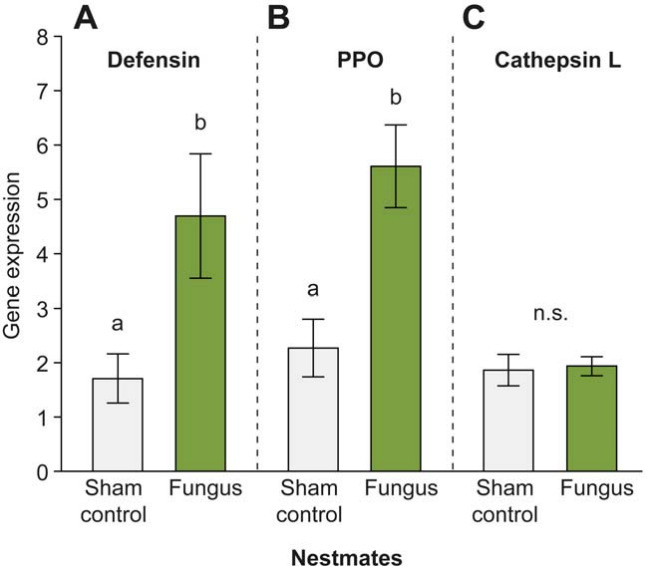
Immune gene expression in nestmate ants. Expression of the immune genes (A) *defensin*, (B) *prophenoloxidase* (*PPO*), and (C) *cathepsin L* normalised to the housekeeping gene *18s rRNA* in nestmates of individuals treated with sham control (light grey) and fungus (light green), after 3 d of social contact. Nestmates of fungus-exposed individuals had significantly elevated *defensin* and *PPO* expression levels compared to nestmates of controls, whereas there was no difference in *cathepsin L* expression. Bars show mean ± SEM (*n* = 7 nestmates of control-treated and 21 nestmates of fungus-exposed individuals for each gene). Different letters indicate statistically significant differences at α = 0.05; n.s., non-significant.

We compared mRNA levels of the three genes in nestmates of fungus-exposed individuals versus nestmates of control-treated individuals on day 3—that is, the first day that we observed an increase in their antifungal activity (Figure 1). After normalising to a housekeeping gene (*18s rRNA*), elevated expression was observed in nestmates of fungus-exposed individuals relative to nestmates of control-treated individuals for both *defensin* and *PPO* (Figure 6; *defensin*: Welch's *t* test; Welch *t* = −2.348, *df* = 26, *p* = 0.032; *PPO*: *t* test; *t* = −2.923, *df* = 26, *p* = 0.007), whereas *cathepsin L* showed no difference (*t* test; *t* = −0.094, *df* = 26, *p* = 0.926). This reveals an active upregulation of immune gene expression in nestmates of fungus-exposed ants and suggests the induction of a specific immune response distinct from immune responses to bacteria (Figure S4; [16]). Similar specific immune upregulation after fungal infection is known to occur in *Drosophila* [77].

**Figure 7:**
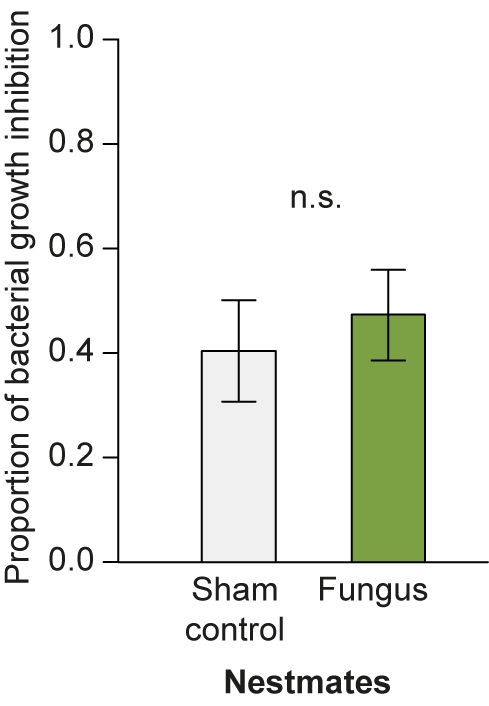
Antibacterial activity of nestmates after social immunisation against the fungal pathogen. The capacity to inhibit growth of the bacterium *Arthrobacter globiformis* did not differ between nestmates of individuals treated with sham control (light grey) and fungus (light green). Bars show mean ± SEM of bacterial growth inhibition standardised to the bacterial growth control (*n* = 10 samples per group, each sample consisting of a pool of five nestmates); n.s., non-significant.

To determine if the observed specificity in our candidate gene approach, which is limited to a small set of genes, reflects specificity at the functional level, we tested the nestmates' capacity to inhibit growth of the bacterium *Arthrobacter globiformis* in an “antibacterial activity assay.” We found that nestmates exhibited similar antibacterial activity for fungus and control treatment (Figure 7; *t* test: *t* = −0.644, *df* = 18, *p* = 0.528), revealing that social immunisation after fungal exposure of the colony is specific and does not lead to a protective effect against bacteria.

### Effects of Active Immunisation Via Low-Level Infections on Colony-Level Epidemiology

**Figure 8:**
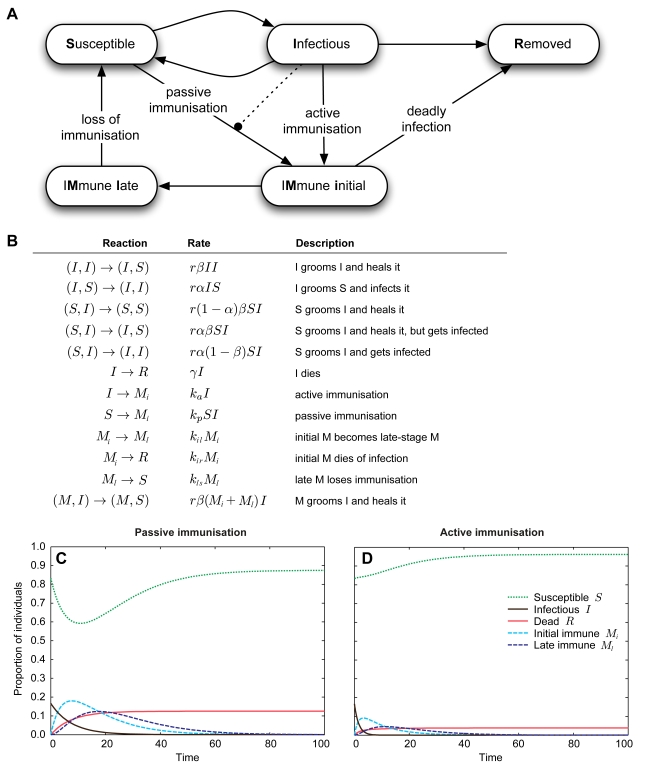
Epidemiological model including two modes of immunisation. Model setup and outcomes. (A) Illustration of the SIRM (Susceptible-Infectious-Removed-iMmune) model, with (B) corresponding state changes and transition rates under which ants change their states. The dotted line in (A) illustrates the influence of infectious individuals (*I*) on the state change rate from susceptible (*S*) to initially immunised (*M_i_*) ants for passive immunisation. (C,D) Model predictions for the proportions of individuals in the different states over time, comparing passive (C) and active (D) immunisation. Passive immunisation allows for a higher number of immune individuals (*M_i_* and entering the *M_l_* state, pale and dark blue dashed lines), whereas active immunisation leads to a faster elimination of the disease (infectious [*I*, black solid line] individuals go to 0) and a lower death rate in the colony (*R*, red solid line), despite the fact that disease spread from the first exposed ants can only occur in the active immunisation scenario. Immunisation is transient so that *M_l_* individuals become susceptible (*S*, green dotted line) over time for both passive and active immunisation.

We developed an epidemiological model to explore the adaptive value and colony-level long-term effects of social immunisation. We compared the effect of active versus passive immunisation in our ant-fungus system by extending classical SIS and SIR (Susceptible-Infectious-Recovered/Removed) models, which describe the progress of epidemics over time using the simplification that the diversity in the population can be reduced to a few states. Possible states in SIR models include individuals *susceptible* to the disease outbreak (*S*), *infectious* individuals (*I*), and *recovered* or *dead* individuals (*R*; [78],[79]). We included an active or passive immunisation mechanism by constructing a SIRM (Susceptible-Infectious-Removed-iMmune) model, in which ants can take five different states. Healthy nestmates are defined as susceptible (*S*) individuals, pathogen-exposed individuals as infectious (*I*) ones, and individuals dying from the disease are removed (*R*) from the model. Successful immunisation (by active or passive immunisation) leads to *initially immune* (*M_i_*) individuals that may persist to create *late-stage immune* individuals (*M_l_*; Figure 8). We describe the mean number of ants in each state by ordinary differential equations (ODEs; for details, see Text S2). We have thereby chosen a simple approach focusing on the comparison of active versus passive immunisation, but not taking into account spatial effects on epidemiology in societies that have been modelled elsewhere by cellular automata [27],[80],[81] or pair-wise approximations models [82].

Ants can change their state by social interactions with each other and depending on their infection state (Figure 8A,B). Allogrooming reduces the fungus load of infectious (*I*), changing them to susceptible (*S*), but at the same time can increase the fungus load of the susceptible individuals (*S*), changing them to infectious (*I*). Active immunisation can occur when individuals receive a low-level infection and actively build up immunity, changing from infectious (*I*) to immune (*M_i_*) with a given active immunisation rate. Under passive immunisation, susceptible (*S*) individuals change directly to the immune state (*M_i_*) with a passive immunisation rate when receiving antimicrobial substances from infectious (*I*) individuals. Under the active immunisation scenario, initially immune ants (*M_i_*) may then either die (*R*) if infection levels are too high and lead to the disease or enter into the later stage of immunity (*M_l_*). Under passive immunisation, all initially immunised individuals become late-stage immune. Late-stage immune ants (*M_l_*) can then lose their immunisation and become susceptible individuals (*S*; see Figure 8A,B and Text S2). Each transition is governed by a transition rate, which in total were fixed to similar ranges in order to allow easy model comparison. The following qualitative results did not depend on the precise rate values, so that we report only representative outcomes of our simulations in Figure 8C,D.

We found that more individuals typically reach the immune state (*M_i_*, and turn into *M_l_*) after passive immunisation (Figure 8C), as a single infectious individual may immunise multiple susceptible nestmates, whereas actively immunised ants need to first be in the infectious state themselves. Yet we found that infections die out (*I* becomes 0) more quickly under active immunisation (Figure 8D), leaving only a very small reservoir for individuals to become immunised. Moreover, active immunisation leads to a lower number of dead individuals (*R*). This is despite the fact that contraction of disease through pathogen transfer can only occur in the active route (with a risk of dying similar to our experimental outcome). Increasing this risk leads to higher death rates and lower immunisation in a linear relationship (simulations not shown). Taken together, active immunisation via pathogen transfer seems beneficial, as it allows more rapid disease elimination and produces lower death rates in colonies, except if the pathogen requires only a very low exposure dose to establish lethal infections in its host.

### Conclusion

In this study, we identified active immunisation as the underlying mode of social group-level immunisation in ant societies after fungal exposure of single individuals. Social contact to a fungus-exposed individual led to low-level infections in the majority of previously naive nestmates (Figures 3, S1, S3) and to a higher capacity to inhibit fungal growth (Figure 1). We found that these low-level infections per se, even in the absence of social contact, are necessary and sufficient to explain the increased antifungal activity of nestmates (Figure 4). We found no evidence for visual or volatile chemical cues acting as additional trigger signals for the immune stimulation of the nestmates. Furthermore, neither ant behaviour (Figure 2) nor physiology (Figure 5C,D) gave an indication for passive nestmate immunisation via transfer of antimicrobials from either exposed ants or their nestmates to the other group members. Finally, experimental elimination of the active route resulted in the absence of protective antifungal activity in nestmates (Figure 5A,B). The increased immune activity of nestmates of fungus-exposed individuals correlates with an increased expression of immune genes such as the antimicrobial peptide *defensin* and the enzyme, *prophenoloxidase* (*PPO*, Figure 6A,B), which both have known antifungal properties [55],[83]. *Cathepsin L*, a lysosomal protease rather involved in antibacterial and antiviral responses ([75],[76]; Figure S4), was not expressed at higher levels in nestmates of fungus-exposed compared to control-treated ants (Figure 6C). In addition to the specific immune gene upregulation revealed by our candidate gene approach, we also found in a functional assay that nestmate immunity is not generally increased, but acts against *Metarhizium* fungus (Figure 1) and not *Arthrobacter* bacteria (Figure 7). Precisely how specific social immunisation is at both the functional and gene expression levels remains to be addressed, and will be facilitated by the emerging genomic information on ants and other social insects [84]–[87].

To our knowledge, our study provides the first mechanistic explanation for the phenomenon of reduced susceptibility of nestmates after social contact to a fungus-exposed individual, that is, social immunisation, described for both ants [18] and termites [17]. Whether group-level immunisation in termite societies follows the same principle as in *Lasius* ants remains to be shown. Interestingly, our study on fungal exposure contrasts with findings of the suggested mechanisms of social immunisation of ants after bacterial exposure, where transfer of antimicrobial substances from the exposed individual via social feeding seems to elicit protection of nestmates [16]. We suggest that distinct infection modes of bacterial and fungal pathogens underlie these differences. Bacterial infections typically occur via oral uptake [39], so that bacteria-exposed individuals do not carry socially transferable spores on their cuticle, as is the case with entomopathogenic fungi. Moreover, the long delay between exposure and infection is not common in bacterial infections, allowing for faster production of immune effectors in the exposed individuals and an earlier potential onset of immunisation.

Social immunisation may not be limited to the highly eusocial insect societies but could similarly occur in other societies or at the family level. If also detected in vertebrates, the underlying mechanisms may be very different, as vertebrates have the additional adaptive/acquired immune component and do not rely solely on the innate immune system that characterises invertebrate immunity [1],[21]. Humans have used the intentional transfer of low-level infections—referred to as “variolation” or “inoculation”—in an attempt to fight smallpox and frequently succeeded in creating long-term protection against this otherwise often deadly disease [62],[63]. In humans, the technique was later replaced by less risky immunisation with attenuated strains as soon as these became available [88], but variolation is still used for, for example, poultry disease management [64]. It is still unclear whether acquiring the protective low-level infections in ants is also an active strategy or, rather, an unintentional byproduct of social contact similar to “contact immunity” occurring in human societies, for example, after live strain polio or smallpox vaccination, where vaccinated individuals became spreaders and vaccinated their family members [89],[90]. It is interesting that allogrooming by the ants is not restricted to single individuals, which would be a good strategy to avoid infecting the whole colony, but is rather performed by many colony members, all of which pick up a low-level infection. This may hint at social immunisation by low-level infections being an adaptive evolutionary strategy.

Our epidemiological modeling indeed suggests that active immunisation is a beneficial strategy for ant colonies, as it allows for faster disease elimination and therefore leads to lower death rates than passive immunisation would. This is particularly true if exposure to low pathogen levels confers a low risk of mortality, as is the case with *Metarhizium* fungus, which requires relatively large doses to elicit a deadly course of disease. We therefore predict that social transfer of pathogens with higher infectivity [91] would not be an advantageous strategy for societies. A comparative analysis of mechanisms employed by social insects against pathogen types differing in their virulence and transmission would thus be highly interesting. Moreover, it seems likely that active immune stimulation following low-level infections may induce individual immune priming and, thereby, a longer lasting protection of colony members than if they simply received immune effectors. The long-lived societies of social insects [43] are at especially high risk of re-encountering the same pathogens multiple times during their lifespans [21], and could greatly benefit from a persistent, rather than transient, social immunisation, particularly against common pathogens such as the fungus *Metarhizium*. To fully understand long-term epidemiological dynamics at the society level it will be indispensable to learn more about the mechanisms involved at the individual level—for example, to better understand if immune priming plays a role in social immunisation.

## Materials and Methods

### Host Ants

The unicolonial ant species *Lasius neglectus* [92],[93] was sampled from four populations (Jena, Germany; Volterra, Italy; Seva and Bellaterra, both Spain; for details on sample locations, see [94]) and reared in the laboratory as described in Ugelvig and Cremer (2007) [18]. Behavioural observations were performed on workers collected in 2006 from all four populations, whereas all further experiments used *L. neglectus* workers collected in 2008 from Jena, Germany. Ants were kept at a constant temperature of 23°C with 75% humidity and a day/night cycle of 14 h light/10 h dark during the experiments. Experiments were performed in petri dishes with a plastered floor and 10% sucrose solution as food.

### Fungal Pathogen

We used the entomopathogenic fungus *Metarhizium anisopliae* var. *anisopliae* (strain Ma 275, KVL 03-143; obtained from Prof. J. Eilenberg, Faculty of Life Sciences, University of Copenhagen, Denmark) to expose the ants in our experiments. To determine inhibition of fungal growth by ant material (antifungal activity assay, see below) and the transfer of conidia to the cuticle of nestmates traced by fluorescence microscopy, we used the RFP (Red Fluorescent Protein) labelled strain 2575 ([95]; obtained from Prof. M. Bidochka, Brock University, Canada). For exposure of ants, we applied the fungal conidia (conidiospores)—that is, the dispersal form that is produced in a natural infection cycle from dead insect cadavers [30]—on the ants, whereas we used blastospores—that is, a single cell spore stage produced inside the body of the infected host [30],[52]—for measuring the antifungal activity. Multiple aliquots of conidia of each strain were kept at −80°C and were grown on malt extract agar at 23°C for 2–4 wk prior to each experiment. Conidia were harvested by suspending them in 0.05% Triton X-100 (Sigma) and stored at 4°C for a maximum of 3–4 wk. All conidia suspensions had a germination rate of >98% as determined directly before each experiment. We produced liquid cultures of blastospores following an adjusted protocol by Kleespies and Zimmermann (1994) [96], though growing the spores at 23°C. Blastospores were harvested by sieving them through a sterile 41 µm nylon net filter (Merck Millipore).

### Fungal Exposure of Ants

We exposed individual ant workers by applying a 0.3 µl droplet of a suspension of 10^9^ conidia/ml in 0.05% Triton X solution (fungus treatment), which corresponds to the lethal dose (LD) 50 for isolated ants. To obtain *low-level infections* in the same order as those picked up by the nestmates during social contact (as confirmed by comparison of internal infection load of the socially transferred and directly applied group), we exposed the ants to 0.3 µl of a 10^5^ conidia/ml suspension (LD_2_) and kept them isolated. For the sham control, we treated the ants with a 0.3 µl droplet of a 0.05% Triton X solution only. Subsequently, the ants were dried on a piece of filter paper for several minutes.

### Experimental Setup

We grouped six workers (1 treated individual and 5 naive nestmates, to be distinguished by colour marking [Edding 780]) and three larvae of *L. neglectus* in a petri dish (Ø = 5.5 cm) with a dampened plaster floor and a piece of filter paper (1×1 cm) moistened with 10% sucrose solution as food supply. The treated individual received either a sham control or a fungus treatment as described above. Our experimental setup is equivalent to the experiment described in more detail in Ugelvig and Cremer (2007) [18], which either led to a social immunisation of nestmates (fungus treatment) or not (sham control) after 5 d of social contact. We used this setup for observations of ant-ant interactions, obtaining physiological immune measures and conidia transmission analysis, yet made some measurements already after 1, 2, or 3 d of social contact.

We changed this general setup for two experiments. First, to determine if signal transfer alone may be sufficient to elicit social immunisation in nestmates, we prevented direct social contact between the treated ant (*n* = 10 for sham control and fungus treatment, respectively) and its nestmates. This was done by keeping the treated individual in a plastic tube (200 µl, Ø of opening = 0.7 cm, containing cotton wool moistened with 10% sucrose solution), attached to the main petri dish, but separated by a double-layered nylon mesh (mesh size 20 µm). The setup prevented direct physical contact yet allowed exchange of visual or volatile chemical signals. After 3 d, nestmates were frozen and subjected to the antifungal activity assay as described below. In a second setup, we excluded both signal and pathogen transfer from the exposed individual to its nestmates occurring in the first 2 experimental days, only allowing for potential later exchange of antimicrobial substances. To this end, we removed the exposed individual 2 d after fungal exposure from its “early nestmates” and placed it with “new nestmates” (Figure 5A), the latter being tested for their antifungal activity after 3 d with the treated individual (*n* = 10 replicates for sham control and fungus treatment, respectively). The new nestmates therefore only had contact to an exposed nestmate after conidia had firmly attached to the host's cuticle, and no longer could be transferred to nestmates (as experimentally confirmed by absence of colony forming units [CFUs] in the new nestmates, see below). When removing the treated individual, we added five new nestmates to the five early nestmates (Figure 5B) to test if early nestmates may transfer immunity to the new nestmates in the form of antimicrobial substances. New nestmates were frozen after 3 d of social contact to the early nestmates of either the control-treated or fungus-exposed individual, and their antifungal activity measured as described below.

### Behavioural Observations of Ant-Ant Interactions

All workers in the observed ant groups were individually colour marked. We then conducted 10 daily behavioural scan samples for each individual in each of six ant nests (replicates) from each of the four study populations (total *n* = 24 ant groups per treatment, i.e. 288 ants) over the 5 d of the experiment (as described in [18]). We were interested in the behavioural interactions between different individuals, which we analysed separately for interactions between the treated individual (total interactions *n* = 240 per treatment) and its nestmates and among nestmates only (total interactions *n* = 480 per treatment). The following types of interactive behaviours could be recorded: antennation (recognition behaviour), allogrooming (mutual cleaning of the body surface), and trophallaxis (exchange of regurgitated liquid food; [38]). For statistical analysis of the behavioural data, see the statistics section below.

### Antifungal and Antibacterial Activity Assay

We developed a sensitive antifungal and antibacterial assay (MS, unpublished) that reveals the antimicrobial activity of ant tissue via the growth inhibition of a pathogen culture (as reduced absorbance in a spectrophotometer) compared to a pathogen growth control without an ant sample. For each assay, we first determined the required ratio of pathogen, ant sample, and buffer to be in the linear range of the growth curve in which antimicrobial activity could be detected. We measured growth inhibition against blastospores of *M. anisopliae* by using either complete ants (*n* = 10 replicate samples for each group), specific ant body parts (gaster cuticle and thorax; *n* = 6 replicate samples for each group), or the trophallactic droplet (*n* = 10 replicate samples for each group) of treated ants (sham control and fungus treatment) and their respective nestmates. Most measurements were taken 3 d (i.e., 72 h) after treatment of the single individual. Nestmates of control and exposed ants were also analysed on day 5 (i.e., 120 h) after treatment. Bacterial growth inhibition against vegetative cells of *A. globiformis* was determined for the nestmates of fungus-exposed and control-treated individuals (*n* = 10 replicates each). In all cases, the body parts or exudates from five individuals were pooled to obtain a single replicate sample. Both antifungal and antibacterial activity was determined as the reduction of either *M. anisopliae* fungal blastospore or *A. globiformis* bacterial vegetative cell growth, measured as absorbance in a spectrophotometer (SpectraMax M2e, Molecular Devices, similar to [97],[98]), after incubation of ant samples with the fungal or bacterial suspension. For detailed information, see Text S1, and for statistical analyses, see below.

### Detection of Fluorescently Labelled Conidia on the Ants' Cuticle

We set up 15 experimental groups each consisting of five nestmates and one individual exposed to RFP-labelled conidia. After 2 d of social contact all ants were removed and frozen at −20°C. The cuticles of three random nestmates per group—that is, 45 nestmates in total—and cuticles from the 15 directly exposed individuals were examined for the presence of RFP-labelled conidia using a fluorescence microscope (Leica MZ16 FA; Software: Leica Application Suite Advanced Fluorescence 2.3.0; Filter Cube: ET DsRed). Each ant was screened for the presence of conidia for a maximum duration of 30 min. In addition we checked the cuticle of 15 naive ants as negative control using the same method. We did not detect any structures resembling RFP-labelled conidia on any of the naive ants.

### Determination of Fungal Infection Loads by Colony Forming Units (CFUs)

We exposed 30 ants, kept them in individual petri dishes, and randomly assigned them to either of the three groups (*n* = 10 ants each): ants that were frozen (−20°C) after 1, 3, or 5 d post-exposure. On day 1 post-exposure 10 of 10 ants were alive, 3 d post-exposure 8 of 10 ants survived, and 5 d post-exposure 4 of 10 ants survived. In addition, we set up 21 experimental groups, each consisting of five nestmates and one fungus-exposed individual, which were also frozen (in equal numbers) 1, 3, or 5 d post-exposure. None of the nestmates had died at this time point.

All individually kept, directly exposed ants (i.e., 10 per day) and two randomly chosen nestmates per experimental group (i.e., 14 per day) were surface-sterilised in ethanol and sodium hypochlorite (as described in [18]) to destroy all fungal material on the cuticle prior to dissection under a stereomicroscope (Leica S6E). For each ant, all contents of the gaster (abdomen) without the cuticle were removed and dissolved in 30 µl of Triton X. The body contents were then plated on selective medium agar plates (containing: chloramphenicol 100 mg/l, streptomycin 50 mg/l, dodin 110 mg/l) and kept at 23°C. After 2 wk of cultivation, the number of colony forming units (CFUs) per plate was determined. We identified CFUs as pure *M. anisopliae* cultures by morphological fungal determination and amplification of specific *M. anisopliae* genes by PCR (see Text S1). For statistical analysis, we used both presence/absence of CFUs for each individual and the number of CFUs growing out of infected ants (for details, see statistical analysis section below).

For method development, we performed the following negative controls: (a) 15 completely untreated ants and (b) 15 ants that were exposed to conidia but were surface-sterilised after 3 h (i.e., before the fungus could penetrate the cuticle and reach the inside of the ant). We did not detect any fungal growth from these 30 ants. Moreover, we could confirm that pathogen transfer did not occur towards the new nestmates of either directly exposed ants or early nestmates (*n* = 14 replicates each).

### Determination of Nestmate Death by Fungal Infection

We set up 30 experimental groups consisting of five nestmates and one fungus-exposed individual each. After the 5 d of social contact to the exposed individuals, each nestmate was isolated in a single petri dish for another 12 d. During the whole experimental period of 17 d, the survival of nestmates was checked daily. Dead nestmates were surface-sterilised as above and put on moist filter paper in a petri dish at constant temperature, 23°C. Cadavers were checked for a period of 3 wk for the growth of *M. anisopliae*.

### Septic Injury

The bacterium *Bacillus thuringiensis* (strain NRRL B-18765, obtained from the permanent strain collection of the Northern Research Laboratory, U.S. Department of Agriculture, Peoria, Illinois, USA) was precultured in LB medium and grown to an OD_600_ of 0.1. We centrifuged 1 ml of the suspension at a speed of 3,000× g for 5 min and discarded the supernatant to obtain a concentrated bacterial pellet as in [99]. Ants were immobilized and pricked ventrally between the 2^nd^ and 3^rd^ gaster sternite with a sterilized needle (minutien needles, Sphinx V2A 0.1×12 mm, bioform) dipped in either LB medium (sham control) or the concentrated bacterial pellet (*n* = 10 ants per treatment, replicated three times; i.e., total *n* = 30 ants per treatment). The ants were frozen for gene expression analysis 12 h after pricking.

### Immune Gene Expression

Ants were analysed either individually (nestmates of *Metarhizium*-exposed ants) or in pools of 10 ants (bacterial septic injury) by qPCR for gene expression of three immune genes and the housekeeping gene, *18s rRNA*. For immune genes, we chose the antimicrobial peptide *defensin* [68],[69], the enzyme *prophenoloxidase* (*PPO* [72],[73]), and the lysosomal protease *cathepsin L* [74],[76]. For details of the procedures on RNA extraction, cDNA preparation, and qPCR, please see Text S1 and the statistical analysis section below.

### Statistical Analyses

We always tested the distributions underlying our data and chose the corresponding tests. If data were not normally distributed even after transformation, we applied models with specified error structures or non-parametric tests. Reported *p* values are two-sided. All statistical analyses were carried out in IBM SPSS Statistics version 19.0 or Sigma Stat 3.5 (Systat Software Inc.). All figures are based on raw data.

For the behavioural observations, we first analysed all behaviours overall over the 5 experimental days. Due to the nature of the data (overdispersed count data), generalised linear models (GLM) with negative binomial errors and a log link function were employed using the following factors: *treatment type* (fungus treatment versus sham control), *ant pairing* (treated-nestmate versus nestmate-nestmate), and the interaction between them. As neither nests within populations nor populations behaved differently, they were not included in the final models. We give the likelihood ratio (LR) χ^2^ to test if our overall model explains the data better than a model with only the intercept. As we detected significant differences for allogrooming, we performed a second test to analyse the effect of time in the interactions between treated individuals and their nestmates for the two treatment types separately (*n* = 240) using a GLM with repeated measures. Simple contrasts with day 1 as reference were employed to test the differences between day 1 and the succeeding days (Figure 2B).

For statistical analysis of the antifungal and antibacterial activity, the absorbance values (optical density) of the different treatment groups were compared by one-way ANOVAs or *t* tests as data were normally distributed or could be transformed to obtain normality. For the antifungal activity of nestmates of exposed versus control nestmates, we applied a GLM to analyse the effects of *treatment type* (fungus treatment versus sham control) and *time* (day 3 versus day 5 post-treatment), as well as their interaction (Figure 1).

For analysis of pathogen load, we compared directly exposed and nestmate ants for (a) the proportion of individuals that were infected (i.e., showed at least a single CFU; Fisher exact test) and (b) the number of CFUs in the individuals that showed an infection (Mann Whitney U test; Figure 3). As the experimental grouping did not influence the number of CFUs found in nestmates from the same ant group, this factor could be excluded from statistical analysis comparing treated individuals and nestmates (GLM with negative binomial errors, LR χ^2^ = 112.362, *df* = 34, *p* = 0.000; Replicate, Wald: χ^2^ = 21.273, *df* = 17, *p* = 0.214).

Gene expression analyses were run in two to three technical replicates. Normalised gene expression values (the average of technical replicates, standardised to the housekeeping gene) were either *a priori* normally distributed or could be normalised by transformation and were analysed using *t* test or—in the case of unequal variances between groups (*defensin*, Figure 6A)—Welch's *t* test for unequal variances [100].

### Epidemiological Model

We applied ordinary differential equations (ODE) to extend classical SIR modeling (Susceptible-Infectious-Removed) with an immunised state to a SIRM model (Susceptible-Infectious-Removed-iMmune), in which the immune individuals were further separated into an initial and a late phase of immunity. See Figure 8A,B for the model and how we calculated state changes and Text S2 for model construction and simulations.

## Supporting Information

Text S1 Experimental protocols: antifungal activity assay, antibacterial activity assay, *Metarhizium* specific PCR, immune gene expression.
(DOC)

Text S2 Epidemiological model: basic SIR model, extended SIRM model, simulations.
(PDF)

Figure S1 Determination of conidia on fungus-exposed ants and their nestmates by fluorescence microscopy. Occurrence of fluorescence-labelled (RFP) *Metarhizium* conidia on the cuticle of directly fungus-exposed individuals (A–C) and their nestmates (D–F) 2 d after exposure of the former. Conidia were found on the cuticle of all directly fungus-exposed individuals in high numbers (always 10+ conidia) and on 37% of the nestmates, usually in low amounts (1–10 conidia). In the directly exposed individuals, conidia were often located at sites that are probably difficult to reach via allogrooming and/or self-grooming like the antennal grooves (A), joints of the legs (B), or the back of the head (C), whereas, in nestmates, conidia were mostly found on exposed body parts that are likely to touch other nestmates during social interactions like the antennae (D) or legs (E,F).
(TIF)

Figure S2 Confirmation of identity of fungal infections as *Metarhizium* by PCR. We used *M. anisopliae* specific primers (Text S1) to genetically confirm whether colony forming units (CFUs) from dissected body contents of the ants (see Figure S3) were truly *M. anisopliae* or a contaminant fungus. Lanes 1 and 2 contain positive controls (PCR product of DNA extracted from *Metarhizium anisopliae*). Lanes 3 to 5 represent PCR products obtained from DNA of CFUs grown from the body contents of nestmates of a fungus-treated individual. Lanes 6 and 7 are negative controls (PCR product from DNA extracted from *Beauveria bassiana*). The fact that our samples amplified bands of the same length as the positive controls, whereas our negative controls showed no amplification by the *M. anisopliae* specific primers, confirmed that the fungus growing on the selective medium agar plates was indeed *M. anisopliae*.
(TIF)

Figure S3 Fungal growth from dissected body content of directly fungus-exposed ants and their nestmates. Growth of colony forming units (CFUs) of the fungus *M. anisopliae* on agar plates containing the dissected gaster content of (A) directly fungus-exposed ants and (B) their nestmates at different times after fungal exposure of the treated ant. Fungal growth was not yet detected within the first 24 h (day1), but occurred frequently on days 3 and 5 after exposure of the treated ant. On both days, nestmates showed lower numbers of CFUs than directly exposed ants. See main text and Figure 3 for quantitative analysis.
(TIF)

Figure S4 Immune gene expression after bacterial septic injury in ants. Expression of the immune genes (A) *defensin*, (B) *prophenoloxidase* (*PPO*), and (C) *cathepsin L* normalised to the housekeeping gene *18s rRNA* in individuals pricked with sham control (LB medium, light grey) and the bacterium *Bacillus thuringiensis* (BT, dark blue). After 12 h, bacteria-exposed individuals had significantly elevated *cathepsin L* expression compared to sham controls, whereas there was no difference in *defensin* or *PPO* expression. Bars show mean ± SEM (*n* = 3 independent experiments, each experimental sample containing cDNA from 10 ants per treatment). Different letters indicate statistically significant differences at α = 0.05; n.s., non-significant.
(TIF)

## Abbreviations

CFU: colony forming unit
GLM: general linear model
LR: likelihood ratio
PPO: prophenoloxidase
RFP: red fluorescent protein
SIRM model: epidemiological model: Susceptible-Infectious-Removed-iMmune
