# Supplementary material for: Social Transfer of Pathogenic Fungus Promotes Active Immunisation in Ant Colonies
Source: PLoS Biol. 2012 Apr 3;10(4):e1001300. doi: 10.1371/journal.pbio.1001300 (PMC3317912; doi:10.1371/journal.pbio.1001300)
Supplement: Text S1 — Experimental protocols: antifungal activity assay, antibacterial activity assay, Metarhizium specific PCR, immune gene expression. (DOC) [file pbio.1001300.s001.doc]

Supporting Information (Konrad et al.):

**Text S1: Experimental Protocols**

**Antifungal activity assay**

We analysed 10 replicates (each replicate containing a pool of 5 individuals) of a) nestmates of both fungus-exposed and sham-treated individuals collected after either 3 or 5 days of social contact to the treated ant (total number of individuals: *n* = 200), b) individuals directly exposed to the sham control or a low or high dose of fungal pathogen (*n* = 150), c) nestmates that were prevented from direct contact to the fungus-exposed and sham-treated individuals (*n* = 100) and, d) 'new nestmates' of fungus-exposed and sham-treated individuals as well as 'new nestmates' of 'early nestmates' of fungus-exposed and sham-treated individuals (*n* = 200). For each sample, five whole ants were crushed together in liquid nitrogen using a sterile pestle and dissolved in 50 µl of phosphate buffered saline (PBS). The suspension was then vortexed and centrifuged (Eppendorf 5424 R; at 4°C) for five minutes at 6000 x g, after which time 20 µl supernatant was removed, mixed and centrifuged again for five minutes at 6000 x g. Subsequently 10 µl supernatant was removed and stored until analysis of the antifungal activity (see below) at -20°C.

In addition we froze and dissected 30 fungus-exposed and 30 sham-treated ants under a stereomicroscope (Leica S6E) to determine the antifungal activity of isolated thoraxes and cuticles of the gaster (abdomen). For each sample, the body parts of 5 treated ants were pooled (resulting in 6 replicates per body part per treatment), dissolved in 50 µl of PBS, crushed in liquid nitrogen and treated as described above for whole body samples. These two body parts were the only ant samples not showing antifungal activity compared to the growth controls, a result that was consistent between multiple experiments. We also obtained the trophallactic droplets of 50 fungus-exposed and 50 sham-treated ants (following [1]). As we could only retrieve 0.05 µl regurgitate per ant (collected in a capillary coated with Sigmacote®), we pooled the droplets of five ants of each treatment (resulting in 10 replicates per treatment), dissolved them in 1.75 µl PBS and stored them at -20°C prior to use in the antifungal activity assay.

All the obtained samples were subjected to an antifungal activity assay, in which we measured the reduction of fungal growth (measured as absorbance in a spectrophotometer as in [2]), when incubated with a blastospore suspension of *M. anisopliae*. To this end, we loaded the wells of 96-well Half Area microplates (Greiner Bio-One) with Sabouraud Dextrose Broth (SDB, Sigma-Aldrich), blastospores (8 x 10^6^ spores/ml) and ant sample in specific relative proportions. Assays analysing the antifungal activity of nestmates contained 50 µl SDB, 2 µl blastospores and 2 µl ant sample per well. Assays for nestmates of caged individuals, 'new nestmates' and directly treated individuals contained 50 µl SDB, 2 µl blastospores and 3 µl ant sample per well. Assays analysing trophallactic droplets of directly fungus-exposed individuals contained 27 µl SDB, 1 µl blastospores and 2 µl ant sample per well. For each measurement we added 6-10 *spore growth controls* (in which the ant sample was replaced by PBS) and *standards* (in which both ant sample and blastospores were replaced by PBS). The absorbance of each well was measured directly after the setup and after 24 h of agitation on a vortex mixer at 23°C using a spectrophotometer (SpectraMax M2e, Molecular Devices; wavelength: 600nm; 23°C).

To test if our sham control treatment had an effect on the antifungal activity, we performed the same experiment using 10 whole sham-treated ants and 10 whole untreated ants. The antifungal activity of sham-treated ants compared with completely untreated ants was not significantly different (One Way ANOVA, *F*_2,27_ = 26.634, *p* < 0.001; post hoc Tukey’s test: sham-treated *vs* untreated individuals: p = 0.903; spore growth control *vs* sham-treated / untreated individual: *p* < 0.001 for both comparisons).

**Antibacterial activity assay**

Each sample (pool of five nestmates in 200 µl of PBS buffer, *n* =10 replicates for both control and fungus treatment) was crushed in liquid nitrogen. After vortexing the suspension, it was centrifuged for 10 minutes at 600 x g at 4°C. 150 µl of supernatant was transferred to a new reaction tube and centrifuged again at 3000 x g for 5 minutes at 4°C. 100µl of supernatant was stored at –20°C until further processing.

The antibacterial activity of the ant samples was determined as the growth inhibition of the vegetative cells of the bacterium *Arthrobacter globiformes* [3] as compared to the bacterial growth without ant sample (water control). Similar to the antifungal assay above, we determined bacterial growth by measuring absorbance in a spectrophotometer (SpectraMax M2e, Molecular Devices), following a modified protocol by Vermeij and Kertesz [4]. We loaded the wells of 96-well Half Area microplates (Greiner Bio-One) on ice with 90 µl of an *A. globiformes* culture with OD_600_=0.0625 and 10 µl sample of ant sample, respectively water (control). After loading, the whole plate was warmed for 15 minutes at 28°C before the first measure was taken. To ensure aerobic growth conditions, the plate was shaken for 30 seconds every five minutes. Absorbance measures were taken immediately as well as three hours after setup (i.e. during the log phase of the bacterial growth control) at 600nm wavelength at 28°C.

**Specific PCR to confirm the identity of CFUs as *M. anisopliae***

To confirm our morphological fungus determination, we isolated conidia and hyphae from several single CFUs per agar plate (*n* = 9) using a sterile inoculation loop. We also extracted DNA of *M. anisopliae* var. *anisopliae* (strain Ma 275, KVL 03-143) and that of the entomopathogenic fungus *Beauveria bassiana* (strain KVL 01-139) directly from our fungal culture as positive and negative controls, respectively.

DNA was then extracted by the "freeze-thaw" technique (adjusted from [5]) followed by a chloroform / isoamyl alcohol extraction. The fungal material was frozen with liquid nitrogen, crushed using a sterile pestle and heated for 40 sec at 70°C on a heating plate. This procedure was repeated three times. The fungal material was then resuspended in 500 µl buffer (2% CTAB, 0.75M NaCl, 50 mM Tris/HCl pH 8.0, 10 mM EDTA) and incubated for 1h at 65°C / 850 rpm in a thermomixer (thermomixer compact, Eppendorf). Each suspension was then cooled down to 55°C and incubated for 8 h at 850 rpm together with 2 µl Proteinase K (Fermentas). All subsequent extraction-steps were performed at ≤ 4°C. We added 500 µl chloroform / isoamyl alcohol (24:1), mixed the suspension, then centrifuged for 7 min at 16000 x g. The upper phase (containing the DNA) was removed, incubated at -20°C for 30 min with 40 µl sodium acetate and 350 µl isopropyl alcohol, then centrifuged for 35 min at 16000 x g. The DNA pellets were washed three times: first with 300 µl 100% ethanol, then spun for 14 min, at 16000 x g; next with 300 µl 70% ethanol and spun for 7 min at 16000 x g; and finally with 150 µl 70% ethanol, and spun for 7 min at 16000 x g. DNA pellets were dried for 30 min at 23°C and 10 µl PCR water (Sigma-Aldrich) was added.

For each sample, we added 1 µl DNA to a mix containing 1.5 µl dNTPs (Fermentas; 1 mM), 1 µl Taq polymerase (MP; 1 U/µl), 2 µl magnesium chloride (Fermentas; 25 mM), 2.5 µl 10x Taq buffer (Fermentas), 11 µl PCR water and 0.5 µl of each the primers ITSMet and ITS4 (Eurofins; 10 µM), which are specific for *M. anisopliae* [6]. PCR reactions were performed in a thermocycler (Mastercycler epgradient, Eppendorf) with following amplification conditions: 2 min denaturation at 94°C, followed by 30 cycles of 94°C for 30 sec, 50°C for 30 sec and 72°C for 45 sec and a final extension at 72°C for 5 min. For electrophoresis, 2 µl of the PCR products were loaded on 1.5% agarose gels containing 1x TBE buffer and stained with Serva Green (Serva) before visualisation under UV light.

**Immune gene expression**

Individual ants (nestmate experiments) or pools of ten ants (septic injury experiments) were frozen at -80°C then dipped into liquid nitrogen prior to grinding with a micropestle for RNA extraction. Total RNA was extracted using Isol-RNA Lysis reagent (5 Prime) according to manufacturer’s instructions. The purity and concentration of RNA were determined by optical density (OD) measurements on a Nanodrop spectrophotometer, and 500 ng total RNA was used for cDNA synthesis. DNAse-I treatment (Invitrogen) was performed on whole samples prior to reverse transcription. Reverse transcription was performed using PeqGold M MuLV-H-minus (Peqlab) per manufacturer’s instructions. Gene expression analyses of *defensin*, *prophenoloxidase*, *cathepsin L* and *18s rRNA* were performed in 20 μl reaction volumes using KAPA SYBR Fast qPCR master mix (Peqlab) and 0.2 μM each of specific primers (Sigma-Aldrich) on a Bio-rad CFX96 real-time PCR detection system. Samples of cDNA corresponding to 10 ng mRNA were added to the plate in 2 μl volumes and each sample was analysed in duplicate or triplicate wells.

Primers were designed based on sequences of closely related species (for *18s rRNA*, *Camponotus laevigatus*, Genbank ID AY218315.1; for *defensin*, *Lasius austriacus*, Genbank ID EU401747.1; for *prophenoloxidase*, *Apis mellifera* Genbank ID AY242387.2; for *cathepsin L, Bombus impatiens*, Genbank ID XM003489516.1), which were then tested for specificity and amplification efficiency on *Lasius neglectus* cDNA samples by standard PCR. PCR products were sequenced and specific primers for qPCR (amplicon lengths 138 bp, 91 bp, 111 bp, and 109 bp, respectively) were designed based on the *Lasius neglectus* sequences. Primer efficiency was found to be between 92-98% for all primer sets using standard curves of 10-fold dilutions of known input amounts, and primer specificity was monitored based on melt curve analysis following each run. The program used for amplification was the following: 95°C for 3 min, followed by 40 cycles of 3 sec of 95°C denaturation and 30 sec of 60°C annealing/extension.

Primers used for qPCR:

5’-18S-Lasius-2 ATCCCTAGCACGAAGGAGGT

3’-18S-Lasius-2 CGCACGAGATTGAGCAATAA

5’-Def-Lasius-3 AAGAACACCATCGCGCACGTAG

3’-Def-Lasius-3 CTGAGAATGCAGTGAGCAGCGC

5’-PPO-Lasius-1 GGTGCGACTGGCTACTAACG

3’-PPO-Lasius-1 ATCGAGCGTCTGTGCAACC

5’-cathepsin-Lasius-1 GCGAAGGAGTTTATTACGAGCC

3’-cathepsin-Lasius-1 ACCAGCCAGTAATCCTCACCAG

The standard curve method was used to calculate differential expression of mRNA transcripts between ant samples [7]. cDNA was used to construct standard curves so that differences in the amplification efficiencies of the standard curve samples and experimental samples could be minimized [8]. The data is expressed as the abundance of immune gene transcripts normalised to the abundance of *18s rRNA* transcripts for each sample.

**References**

1. Hamilton C, Lejeune BT, Rosengaus RB (2011) Trophallaxis and prophylaxis: social immunity in the carpenter ant *Camponotus pennsylvanicus*. Biol Lett 7: 89-92.

2. Fehlbaum P, Bulte P, Michaut L, Lagueux M, Broekaert WF, et al. (1994) Insect immunity: Septic injury of *Drosophila* induces the synthesis of a potent antifungal peptide with sequence homology to plant antifungal peptides. J Biol Chem 269: 33159-33163.

3. Conn H (1928) A type of bacteria abundant in productive soils, but apparently lacking in certain soils of low productivity. NY State Agric Exp stn Tech Bull 138: 3-26.

4. Vermeij P, Kertesz MA (1999) Pathways of assimilative sulfur metabolism in *Pseudomonas putida*. J Bacteriol 181: 5833-5837.

5. Kuske CR, Banton KL, Adorada DL, Stark PC, Hill KK, et al. (1998) Small-scale DNA sample preparation method for field PCR detection of microbial cells and spores in soil. Appl Environ Microb 64: 2463-2472.

6. Destèfano RHR, Destèfano SAL, Messias CL (2004) Detection of *M. anisopliae* var. *anisopliae* within infected sugarcane borer *Diatraea saccharalis* (Lepidoptera: Pyralidae) using specific primers. Genet Mol Biol 27: 245-252.

7. Pfaffl MW (2001) A new mathematical model for relative quantification in real time RT-PCR. Nucleic Acids Res 29.

8. Peirson SN, Butler JN, Foster RG (2003) Experimental validation of novel and conventional approaches to quantitative real-time PCR data analysis. Nucleic Acids Res 31: e73.
