# Supplementary material for: Social Transfer of Pathogenic Fungus Promotes Active Immunisation in Ant Colonies
Source: PLoS Biol. 2012 Apr 3;10(4):e1001300. doi: 10.1371/journal.pbio.1001300 (PMC3317912; doi:10.1371/journal.pbio.1001300)
Supplement: Text S2 — Epidemiological model: basic SIR model, extended SIRM model, simulations. (PDF) [file pbio.1001300.s002.pdf]

Supporting Information (Konrad et al):

Text S2: Epidemiological model

We constructed a SIRM (**S**usceptible-**I**nfectious-**R**emoved-i**M**mune) model, in which ants can take five different states (Figure 1). First, healthy nestmates are *susceptible* (S) to the disease, whereas pathogen-exposed individuals are *infectious* (I). Infectious individuals carry fungal conidia on their body surface and can die of the disease or spread the disease to their nestmates. In addition, they can also immunise their nestmates by either transfer of conidia leading to an active immunisation, or by transferring antimicrobial substances leading to passive immunisation. Individuals can also die from the disease after infection (*dead*, R for removed), or become *immune* (M_i) after successful immunisation. Initially immunised individuals can then either stay immune (M_l, i.e. late-stage immune), or become susceptible (S) again after some time when immune protection is lost. Here, however, social transfer of the pathogen in higher doses than leading to the protective immunisation may also cause individuals fall sick (M_i to R).

We assumed a large enough population of ants to describe the time-varying amount of ants in each state by a continuous percentage of the total population, denoted by $S(t)$, $I(t)$, $R(t)$ and $M_{i(t)}, M_l(t) \in [0,1]$, respectively. Moreover we assumed random encounters of ants ('mass action kinetics'). We could then describe the mean number of ants using a system of ordinary differential equations (ODEs). This implied specifying the change of each state's concentration as a function of the system state, in combination with a set of rate parameters. We first constructed a model without

immunisation, only with states S, I and R, and extended it later by including initial and immunisation M_i and M_l (Figure 8).

Model without immunisation (SIR). Ants can change their fungal load and thus their state changes during allogrooming, which occurs between individuals with a rate r. We assumed that during allogrooming, the pathogen load of the groomed partner will be reduced, yet the pathogen load of the grooming ant may increase, leading to the following two transition rates: if a susceptible ant grooms an infectious ant, it can get infectious itself with an infection probability α. If an infectious ant is being groomed, the conidia may be removed with a certain success probability β so that an infectious individual becomes a susceptible one. We also included a death rate γ, which implies a state transition from I to the dying state R. Without immunisation, the model is then defined by six state changes, which can be translated into the following ODE model:

Equation Set 1:

$$\frac{dS}{dt} = r\beta I^2 - r\alpha SI + r(1-\alpha)\beta SI - r\alpha(1-\beta)SI$$

$$\frac{dI}{dt} = -r\beta I^2 + r\alpha SI - r(1-\alpha)\beta SI + r\alpha(1-\beta)SI - \gamma I$$

$$\frac{dR}{dt} = \gamma I$$

Here r denotes the allogrooming rate i.e. the number of times a particular ant grooms another ant per time unit, at least one of them being infectious. The equations are derived from the upper six state changes listed in Figure 8B. The four terms

describing the change of S are given by allogrooming events of two infectious ants ($r\beta I^2$), which can result in healing of the groomed ant, or allogrooming of an infectious and one susceptible ant, which can either infect the one ($-r\alpha SI$) or heal the other ($r(1-\alpha)\beta SI$), or do both ($-r\alpha(1-\beta)SI$). These terms add to the change of S and correspondingly reduce I. Moreover, death of infectious ants is included by the linear death term γI. The above system simplifies to

Equation Set 2:

$$\frac{dS}{dt} = r\beta I^2 + r(-2\alpha + \beta)SI$$

$$\frac{dI}{dt} = -r\beta I^2 + r(2\alpha - \beta)SI - \gamma I$$

$$\frac{dR}{dt} = \gamma I$$

Model with immunisation (SIRM). We then included immunisation of ants via two mechanistic routes. *Active immunisation* can occur when individuals survive an infection and actively build up immunity. We thus described active immunisation by success of immunisation during infection i.e. by transition from infectious (*I*) to immune (M_i) with rate k_a. *Passive immunisation*, on the other hand, does not involve an infection of later protected individuals as susceptible nestmates receive immune protection by transfer of antimicrobial substances produced by the infectious individuals. Passive immunisation is thus modelled by a transition from susceptible (*S*) to immune (M_i), dependent with factor k_p on the number *I* of infectious ants with available transferable immune effectors. This results in the extended SIRM-model

illustrated in Figure 8A with state changes shown in Figure 8B. The corresponding ODE model is given by:

Equation Set 3:

$$\frac{dS}{dt} = r\beta I^2 + r(-2\alpha + \beta)SI + r\beta(M_i + M_l)I - k_p SI + k_{ls} M_l$$

$$\frac{dI}{dt} = -r\beta I^2 + r(2\alpha - \beta)SI - r\beta(M_i + M_l)I - (\gamma - k_a)I$$

$$\frac{dR}{dt} = \gamma I + k_{ir} M_i$$

$$\frac{dM_i}{dt} = k_a I + k_p SI - k_{il} M_i - k_{ir} M_i$$

$$\frac{dM_l}{dt} = k_{il} M_i - k_{ls} M_l$$

Clearly, the number of dead and immunized ants is always growing, and the dynamics will reach a steady state. In steady state, all derivatives are set to zero, so I=0 i.e. the disease always dies out. Any other combination of S, R, M_i, M_l concentrations (as long as $S+R+M_i+M_l=1$) is clearly stable if I=0, so there are no discrete steady states, and the system is fully controllable by its initial conditions.

Simulation. We then solved the SIRM model numerically for varying initial conditions and active *versus* passive immunisation. As rates, we first fixed $\alpha = 0.1, \beta = 0.5, \gamma = 0.1, r = 0.1, k_a = 0.5, k_b = 0.5, k_{ir} = 0.01, k_{il} = 0.01, k_{ls} = 0.1\}$ under low initial proportion of infectious *versus* susceptible individuals (low: I=1/6, S=5/6; as in the experimental setups; Figure 8).
