# Supplementary figures and images for: Social Transfer of Pathogenic Fungus Promotes Active Immunisation in Ant Colonies
Source: PLoS Biol. 2012 Apr 3;10(4):e1001300. doi: 10.1371/journal.pbio.1001300 (PMC3317912; doi:10.1371/journal.pbio.1001300)

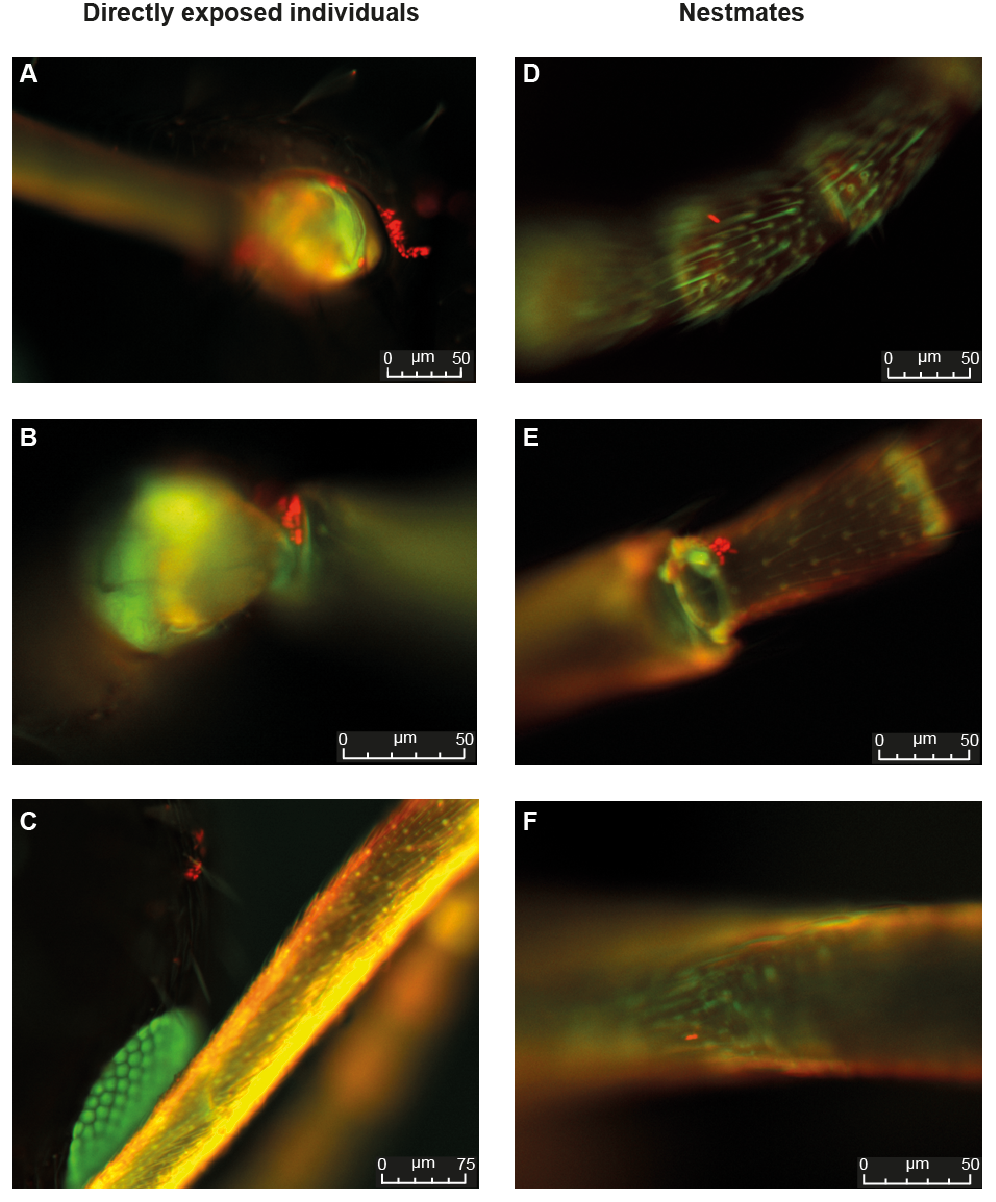

Supplement: Figure S1 — Determination of conidia on fungus-exposed ants and their nestmates by fluorescence microscopy. Occurrence of fluorescence-labelled (RFP) Metarhizium conidia on the cuticle of directly fungus-exposed individuals (A–C) and their nestmates (D–F) 2 d after exposure of the former. Conidia were found on the cuticle of all directly fungus-exposed individuals in high numbers (always 10+ conidia) and on 37% of the nestmates, usually in low amounts (1–10 conidia). In the directly exposed individuals, conidia were often located at sites that are probably difficult to reach via allogrooming and/or self-grooming like the antennal grooves (A), joints of the legs (B), or the back of the head (C), whereas, in nestmates, conidia were mostly found on exposed body parts that are likely to touch other nestmates during social interactions like the antennae (D) or legs (E,F). (TIF) [file pbio.1001300.s003.tif]

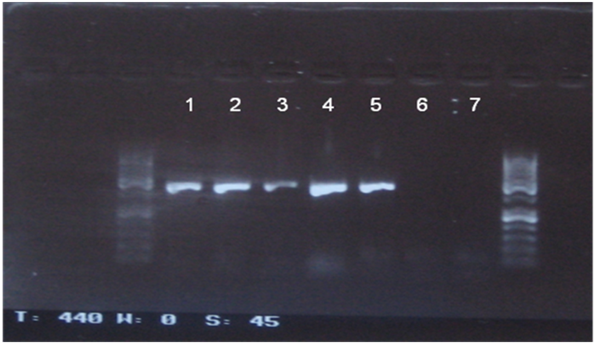

Supplement: Figure S2 — Confirmation of identity of fungal infections as Metarhizium by PCR. We used M. anisopliae specific primers (Text S1) to genetically confirm whether colony forming units (CFUs) from dissected body contents of the ants (see Figure S3) were truly M. anisopliae or a contaminant fungus. Lanes 1 and 2 contain positive controls (PCR product of DNA extracted from Metarhizium anisopliae). Lanes 3 to 5 represent PCR products obtained from DNA of CFUs grown from the body contents of nestmates of a fungus-treated individual. Lanes 6 and 7 are negative controls (PCR product from DNA extracted from Beauveria bassiana). The fact that our samples amplified bands of the same length as the positive controls, whereas our negative controls showed no amplification by the M. anisopliae specific primers, confirmed that the fungus growing on the selective medium agar plates was indeed M. anisopliae. (TIF) [file pbio.1001300.s004.tif]

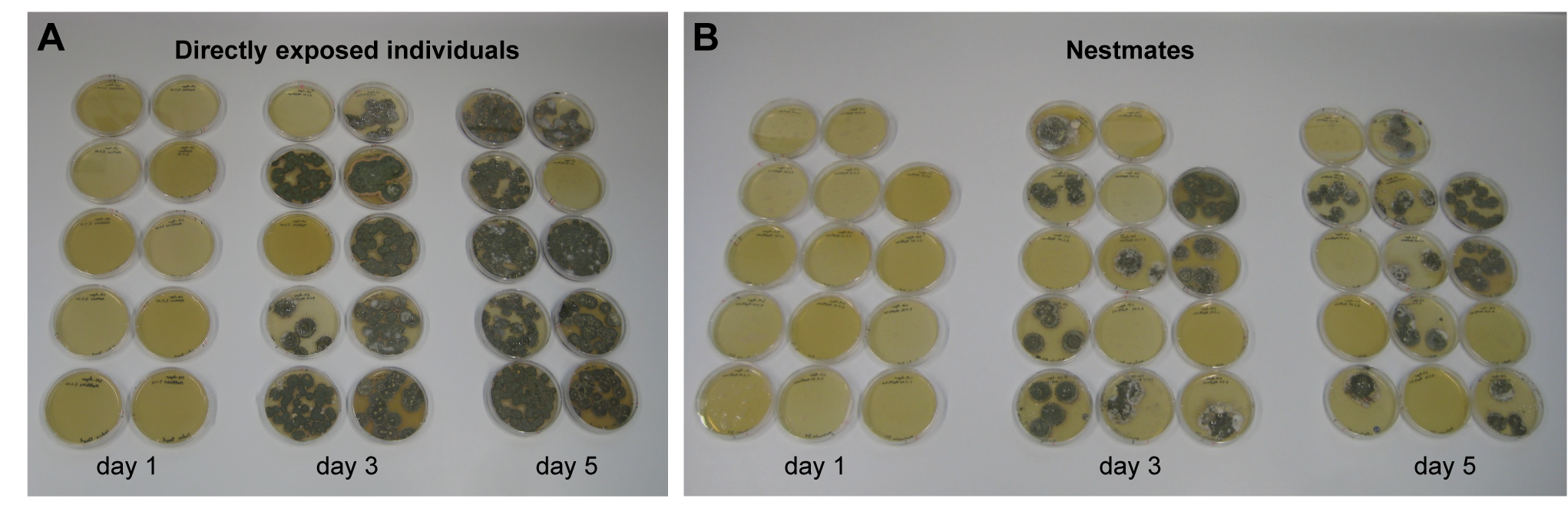

Supplement: Figure S3 — Fungal growth from dissected body content of directly fungus-exposed ants and their nestmates. Growth of colony forming units (CFUs) of the fungus M. anisopliae on agar plates containing the dissected gaster content of (A) directly fungus-exposed ants and (B) their nestmates at different times after fungal exposure of the treated ant. Fungal growth was not yet detected within the first 24 h (day1), but occurred frequently on days 3 and 5 after exposure of the treated ant. On both days, nestmates showed lower numbers of CFUs than directly exposed ants. See main text and Figure 3 for quantitative analysis. (TIF) [file pbio.1001300.s005.tif]

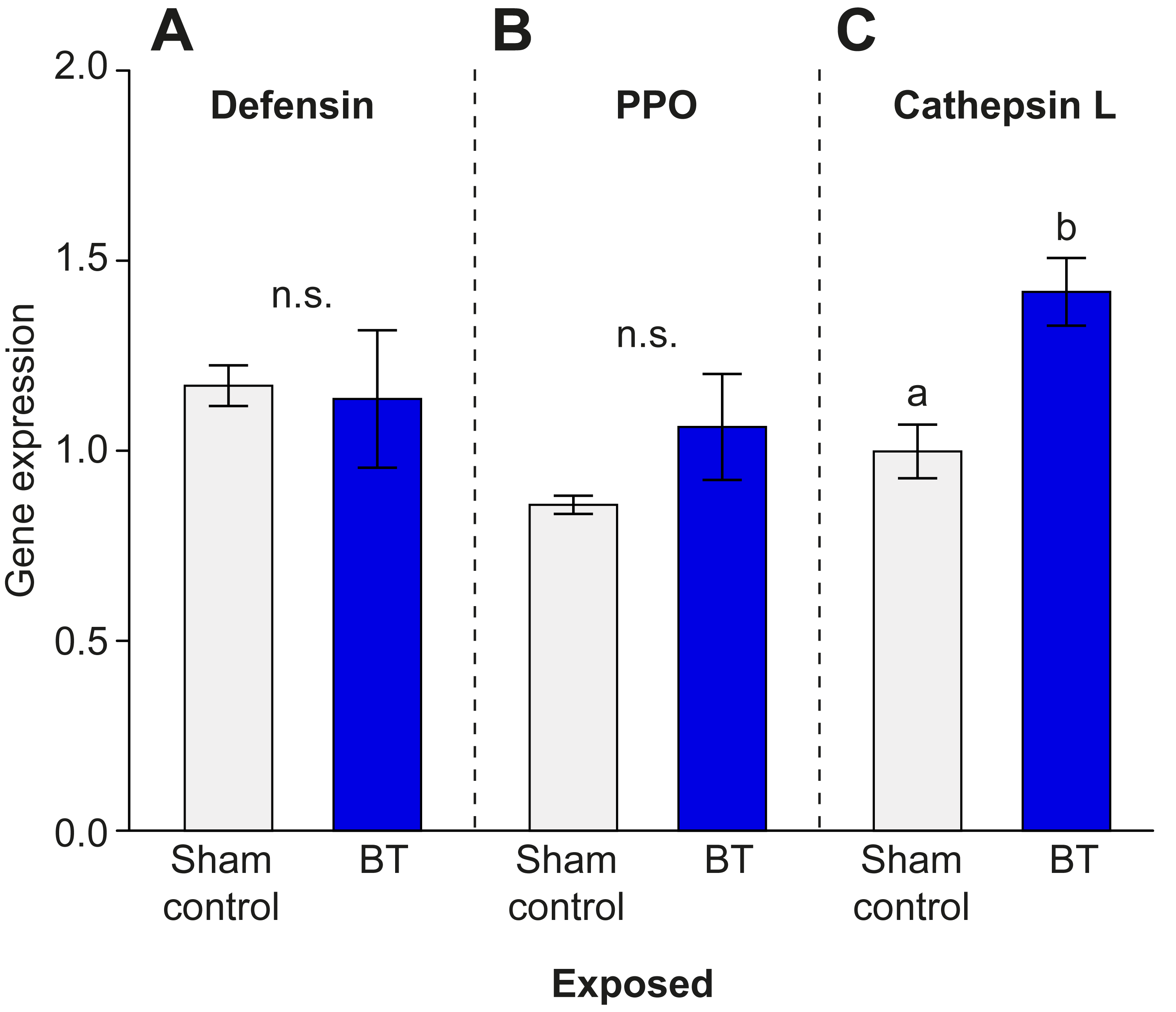

Supplement: Figure S4 — Immune gene expression after bacterial septic injury in ants. Expression of the immune genes (A) defensin, (B) prophenoloxidase (PPO), and (C) cathepsin L normalised to the housekeeping gene 18s rRNA in individuals pricked with sham control (LB medium, light grey) and the bacterium Bacillus thuringiensis (BT, dark blue). After 12 h, bacteria-exposed individuals had significantly elevated cathepsin L expression compared to sham controls, whereas there was no difference in defensin or PPO expression. Bars show mean ± SEM (n = 3 independent experiments, each experimental sample containing cDNA from 10 ants per treatment). Different letters indicate statistically significant differences at α = 0.05; n.s., non-significant. (TIF) [file pbio.1001300.s006.tif]
